# The Need for Robust Critique of Arts and Health Research: Young People, Art Therapy and Mental Health

**DOI:** 10.3389/fpsyg.2022.821093

**Published:** 2022-02-10

**Authors:** Katarzyna Grebosz-Haring, Leonhard Thun-Hohenstein, Anna Katharina Schuchter-Wiegand, Yoon Irons, Arne Bathke, Kate Phillips, Stephen Clift

**Affiliations:** ^1^Department of Musicology and Dance Studies, Faculty of Art History, Musicology and Dance Studies, Paris Lodron University Salzburg, Salzburg, Austria; ^2^Programme Area (Inter)Mediation. Music – Mediation – Context, Interuniversity Institution Knowledge and the Arts, Paris Lodron University Salzburg, University Mozarteum Salzburg, Salzburg, Austria; ^3^Paracelsus Medical University, Salzburg, Austria; ^4^College of Health, Psychology and Social Care, Health and Social Care Research Centre, University Professorial Council, University of Derby, Derby, United Kingdom; ^5^Department of Mathematics, Faculty of Natural Sciences, Paris Lodron University Salzburg, Salzburg, Austria; ^6^Department of Social, Therapeutic and Community Studies, Goldsmiths, University of London, London, United Kingdom; ^7^Canterbury Christ Church University, Canterbury, United Kingdom

**Keywords:** music, arts, youth, psychiatric disorders, health, review

## Abstract

We describe work in progress to conduct a systematic review of research on effects of arts-based programs for mental health in young people. We are at the stage of searching for relevant studies through major databases and screening extant systematic reviews for additional research which meet our inclusion criteria. At this stage, however, concerns have arisen regarding both the quality of existing primary studies and of recently published systematic reviews in this area of arts and health. As a case in point, in this paper we focus on one research report on art therapy with adolescent girls and its inclusion in three systematic reviews. We demonstrate that the reviews fail to undertake a robust critique of the Bazargan and Pakdaman paper and that the paper and reviews are flawed. Drawing on recent criticisms of systematic reviewing, we consider the value of proceeding with our systematic review as initially planned.

## Introduction

Clift et al. (2021) have argued the need for robust critique of research on the social and health impacts of the arts. They consider two recent, scoping reviews of the arts and health literature (Fancourt and Finn, 2019; Fancourt et al., 2020) and document problems associated with a lack of critical perspective on the research included. The positive recommendations drawn in these reviews are called into question, and Clift et al. conclude: ‘it is premature to suggest, as the WHO and DCMS reports do, that the evidence on arts and health provides a secure foundation on which to develop social and health policy. In moving research and practice forward in future, the field must rely on rigorous systematic reviews involving careful quality assessment of both quantitative and qualitative studies’ (p. 13).

Informed by this view, we are currently engaged in conducting a systematic review of controlled studies of creative arts activities/arts therapy for children and young people experiencing challenges to their mental health. A protocol for the proposed review was developed with reference to the latest PRISMA guidelines (Møller and Myles, 2016; Page et al., 2021) and published through PROSPERO.^1^

So far, we have searched major electronic databases and supplemented this approach by cross-checking reference lists in relevant recent reviews. A further tactic has been to use Google Scholar to identify citations of potentially relevant papers in subsequent publications. Our preparatory work, however, has revealed some concerns. Firstly, regarding the questionable quality of published research on the effect of arts-based or creative programmes and therapy for young people with mental health challenges, and secondly, a lack of criticality in recent reviews of this literature.

The aim of this paper is to reflect on what we have found so far, before considering whether to proceed with the planned systematic review. At the time of writing this paper, seven databases have been systematically searched, and two members of the team (KG-H and SC) have independently screened abstracts for relevance. Full text papers have been obtained and organised alphabetically by AKS-W and the first author. The first full text paper to be scrutinised, at the top of the list, is a study of arts therapy for adolescent girls (Bazargan and Pakdaman, 2016). This paper is also included in three recent systematic reviews (Ponomarenko et al., 2017; Cohen-Yatziv and Regev, 2019; Bosgraf et al., 2020). Although Clift et al. (2021) appealed for carefully conducted systematic reviews as the appropriate guide for further developments in research, practice and policy development in arts and health, we will show below these three reviews are far from satisfactory in their treatment of the Bazargan and Pakdaman paper and have additional weaknesses.

In discussing our findings, we will draw on a wider critical literature related to the conduct and value of systematic reviews in medicine and the health sciences.^2^ Ioannidis (2016, p. 486), for example, raises concerns about the ‘mass production’ of systematic reviews and concludes: ‘The production of systematic reviews and meta-analyses has reached epidemic proportions. Possibly, the large majority of produced systematic reviews and meta-analyses are unnecessary, misleading and/or conflicted.’^3^

Møller et al. (2018, p. 520), go further and question whether systematic reviews and meta-analyses are a useful form of research: ‘An evaluation of the landscape of current systematic reviews and meta-analyses suggests that many of them are focused on unimportant questions, many are redundant and unnecessary, a sizeable proportion are flawed beyond repair, and eventually only about 3% of them are both well done and clinically useful.’

Kolaski et al. (2021, p. 10) have conducted a remarkable study of 83 systematic reviews of interventions for children with cerebral palsy, assessed using the AMSTAR-2 appraisal framework,^4^ and conclude that most of the reviews were ‘unreliable.’ They say: ‘… even in recent years when guidelines for sound conduct and reporting of systematic reviews are readily available, most summaries of evidence in systematic reviews about interventions for children with CP continue to be untrustworthy.’

And Negrini et al. (2021, p. 1) in a commentary on the Kolaski et al. paper acknowledge the ‘bleak picture’ they paint, but reaffirm the value of Cochrane style reviews and appeal for: ‘More rigorous literature searches, standardised application of risk of bias tools and analyses and reporting of results that incorporate quality appraisal…’

Fancourt and Finn (2019) based their World Health Organisation scoping review of research in arts and health on ‘over 900 publications (…) of which there were over 200 reviews, systematic reviews, meta-analyses, and meta-syntheses covering over 3,000 studies, and over 700 further individual studies.’ (p. vii) Given the conclusion reached by Kolaski et al. from their scrutiny of systematic reviews of treatments for children with cerebral palsy, and the fact that Fancourt and Finn did not appraise the quality of the systematic reviews they refer to, there is reason to be concerned about the validity of the large and growing number of systematic reviews in arts and health.

In the main body of this paper, we will first summarise the study of art therapy for adolescent girls attending an ‘arts school’ in Tehran, Iran (Bazargan and Pakdaman, 2016) before turning to a discussion of the coverage of this research in three subsequent systematic reviews. We will then offer a critique of the Bazargan and Pakdaman paper, arguing that it should not have appeared in these systematic reviews. We conclude with a broader reflection on the factors which help to explain the production and publication of uncritical systematic reviews.

## Young People, Art Therapy and Mental Health—the Need for Robust Critique

### A Non-evaluative Summary of the Bazargan and Pakdaman Study

Bazargan and Pakdaman (2016) describe the purpose of their study as ‘to determine the effectiveness of art therapy in reducing internalizing and externalizing problems of adolescent girls (14–18 years old)’ attending an ‘arts school’^5^ in Tehran, Iran. ‘Diagnosis of the problems’ was based on a ‘self-completion form’ as part of the ‘Achenbach System of Empirically Based Assessment’ (ASEBA) (for a description see: Achenbach, 2019). ‘Internalizing problems’ involve ‘symptoms’ including ‘anxiety/depression, withdrawal/depression’ and ‘somatoform complaints.’^6^ ‘Externalizing’ problems, in contrast, include ‘rule-breaking and aggressive behaviours.’^7^ Thirty girls who scored at or beyond two standard deviations above the mean on the internalizing ‘symptom scales’ and below +1.3 standard deviations on the externalizing scales were identified as ‘internalizing’ and randomised into either the art therapy or control group in the study. In addition, 30 girls who scored at or above 1.3 standard deviations on the externalizing scales, but below two standard deviations on the internalizing scales, were identified as ‘externalizing’ and similarly randomised to either art therapy or the control condition in a parallel trial. Art therapy consisted of 61 and a half hour sessions in small groups (group sizes not given) led by an art therapist, in which girls painted and then had the opportunity to discuss what they had produced. No details are given of the activity engaged in by the control groups. Following the programme of art therapy, the girls completed the Achenbach self-completion forms for a second time. The authors report that ‘our results showed that Art therapy significantly reduced internalizing problems (…); however, its effect in reducing externalizing problems was not significant (…)’ (p. 51).

### The Inclusion of the Bazargan and Pakdaman Study in Subsequent Reviews

The Bazargan and Pakdaman study is included in three systematic reviews concerned with the potential value of arts therapy for children/adolescents described as ‘vulnerable’ (Ponomarenko et al., 2017), or in five ‘clinical’ categories (Cohen-Yatziv and Regev, 2019) or with ‘psychosocial problems’ (Bosgraf et al., 2020). In each of these reviews, the reported findings from the Bazargan and Pakdaman study are accepted at face value, with little critical appraisal and commentary.

#### Ponomarenko, Yap and Peeran (2017)

The purpose of this review, published in the United Kingdom by the highly respected Thomas Coram Foundation for Children, was to evaluate ‘the existing quantitative evidence base on the impact of delivering music therapy and art therapy to vulnerable children and young people’ (Ponomarenko et al., 2017, p. 10). The substantive focus of the review is on ‘the types of cases most commonly dealt with by art and music therapists working with vulnerable children in the United Kingdom’ (p. 12). Based on experience at Coram, the following list was compiled and used as search terms for the review (p. 15):

- attachment disorders/parent–child bonding issues and/or early relational trauma;
- trauma;
- grief and bereavement;
- anxiety;
- speech, language and communication difficulties;
- behavioural and social interaction difficulties;
- low self-esteem;
- autistic spectrum disorders;
- concentration and learning difficulties; and
- adopted children.

A variety of databases and journals were searched to identify reports published from 2000 onwards for inclusion in the review. The Scientific Maryland Scale (SMS) was used to judge the quality of each study on a five-point scale, with a grade of 5 given to randomised controlled trials.^8^

A total of 896 papers were identified from the initial searches. These were reduced to 430 after removing duplicates and papers that did not match the inclusion criteria. Of the 430 only 61 could be rated against the SMS, and only papers graded at 2 and above were included leaving 51 papers for the review – 14 of which related to art therapy and 37 concerned music therapy.^9^

One of the 14 studies of art therapy included is that reported by Bazargan and Pakdaman (2016). The authors take Bazargan and Pakdaman’s account of their findings at face value and offer no critique, although they do acknowledge that Bazargan and Pakdaman identify limitations to their study. One noteworthy issue in the review is that the study is given an SMS rating of ‘3’ rather than ‘5’ which as a randomised controlled trial it arguably deserves.^10^

#### Cohen-Yatziv and Regev (2019)

The purpose of this systematic review was to assess ‘the effectiveness of art therapy in a wide range of child-aged clients’^11^ (Cohen-Yatziv and Regev, 2019, p. 100). Four electronic databases were searched for quantitative studies on art therapy for children from 2000 to 2017. Thirteen articles were identified^12^ and categorised according to their ‘level of evidence’ as: 1, randomised controlled trials, 2 non-randomised two-group studies and 3 non-randomized one-group studies (p. 101). Studies were also grouped according to the issues addressed through the therapy: trauma, special education and disabilities, no specific diagnosed difficulties, medical condition and juvenile offenders (p. 103–104).

The Bazargan and Pakdaman paper is included in this review under the heading of ‘no specific diagnosed difficulty’ despite the way in which Bazargan and Pakdaman characterise the girls involved as being at the high end of the internalising and externalising distributions. Details of the study are given in Table 3, where it is described as level 1 (p. 108). The study is also briefly mentioned in the text on p. 104 along with two other studies of art therapy with children who had ‘no specific diagnosed condition’ conducted after 2000. They sum up the findings from these studies as showing that: ‘art therapy may help children who are not diagnosed with specific difficulties but are faced with a variety of challenges in life’ (p. 104), but neglect to mention that they involve very different participants: siblings of paediatric stem cell transplant patients; persistent asthma requiring daily treatment and adolescent girls with internalising and externalising problems.

As with the Ponomarenko et al. study, therefore, Cohen-Yatziv and Regev simply take the findings reported by Bazargan and Pakdaman at face value and offer no discussion of the details of their research and its potential problems.

#### Bosgraf, Spreen, Pattiselanno and van Hooren (2020)

Bosgraf et al. (2020) conducted what they characterise as a ‘systematic narrative review’ in order to give an overview of art therapy interventions for children and adolescents with psychosocial problems. Fourteen databases and four electronic journals up to January 2020 were systematically searched. The ‘applied means and forms of expression’, therapist behaviour, supposed mechanisms of change and effects of therapy were extracted and coded. Thirty-seven studies out of 1,299 studies met the inclusion criteria.^13^ These included 16 randomised controlled trials, eight controlled trials and 13 single-group pre–post design studies.

The quality of the studies was assessed by two researchers (LB and KP) applying the ‘Effective Public Health Practice Project (EPHPP) Quality Assessment Tool for Quantitative Studies’ (Thomas et al., 2004)^14^ which has eight categories: selection bias, study design, confounders, blinding, data collection methods, withdrawal and dropouts, intervention integrity and analysis. Independent of each other, they came to an opinion and then discussed their ratings to reach an agreement. Once the assessment was completed, each examined study received a mark ranging between ‘strong,’ ‘moderate,’ and ‘weak.’

Art therapy interventions for children and adolescents varied in terms of materials/techniques used and the extent to which therapist structured the activity. Three forms of therapist behaviour were distinguished: non-directive, directive and eclectic.

The Bazargan and Pakdaman study is described in each of two tables in the paper. Table 1 provides the descriptive details of the study, as was found in the previous two systematic reviews. In addition, the ‘quality assessment’ is described as ‘strong’ but no specific details are given in the paper on how the authors arrived at this rating.^15^

Table 2 reports on the characteristics of the art therapy employed in the Bazargan and Pakdaman study and provide details on the supposed mechanisms through which the activity of painting and discussion served to be therapeutic. Bosgraf et al. characterise these mechanisms, in note form, as follows:

Reveal what they have inside; leads to new activities and enhances experiences; provides an individual with opportunities through which they can freely express their feelings, affections, needs, and knowledge; achieving a feeling of security toward unpleasant memories of a traumatic event; emotions and thoughts are influenced by conflicts, fears, and desires, and painting allows patients to express them symbolically; offering opportunities to regain a sense of personal agency; explore existential concerns; reconnect to the physical body (p. 14).

What is remarkable, however, is that nowhere in the Bazargan and Pakdaman paper do they show that these putative processes took place for the girls in the study. Bosgraf et al. draw on the theoretical, broadly psychoanalytic, account Bazargan and Pakdaman offer in their introduction.

As with the two previous systematic reviews, therefore, we conclude that Bosgraf et al. fail to undertake a careful critical reading of the Bazargan and Pakdaman paper and are content to accept their account of what they did and the results they observed.

### Bazargan and Pakdaman, 2016 – A Robust Critique

Having considered the uncritical treatment of the Bazargan and Pakdaman paper in three recent systematic reviews, we now return to this study and offer a detailed critique. There are at least six problematic features of the Bazargan and Pakdaman study. Taken together, our judgement is that this paper should have been excluded from the systematic reviews considered, and it will not be included in the review we are currently undertaking.

#### Questionable View of Mental Health Challenges and Causal Ontology

An increasing body of literature is critical of the role of psychiatry and psychology in ‘categorising’ and ‘labelling’ people with mental ‘disorders’ (see for example: Gambrill, 2013; Kinderman et al., 2013, 2017; Johnstone et al., 2018). In the Bazargan and Pakdaman paper a questionable view of mental health challenges is reflected throughout in the use of language. For example, the word ‘problem’ is used no fewer than 77 times in the paper, and ‘disorder’ is used seven times. Also, the girls in the study are repeatedly referred to as ‘subjects’ (18 times) and the art therapy activity is described as an ‘intervention’ (11 times).^16^ A further indication of the reification of mental health ‘problems’ in categorising the girls as ‘internalising’ or ‘externalising’ is the fact that no details are given on any aspect of the girls’ circumstances or specific experiences. If the ‘internalising’ girls are depressed, anxious, withdrawn or showing psychosomatic ‘complaints’ it is surely important to understand why these challenges have arisen and what sustains them.

There is also a view inherent in the research design that the art therapy ‘intervention’ has a causal ‘effect’ on the girls’ mental health, without any reference to the role of their active engagement or agency in the process. There is, however, an acknowledgement of the agency of some girls, especially those with so-called ‘externalising’ ‘problems’, in their resistance to taking part in the ‘therapy’ sessions (see the next section).

#### Lack of Trial Registration, Ethical Review and CONSORT Diagram

The first issue to point out about this paper, as a report of two parallel randomised controlled trials, is that there is no indication that the protocol for the study was lodged with a trial register. For virtually all academic journals reporting on trials, this is an essential requirement for publication,^17^ although clearly it was not a requirement to publish in the *Archives of Iranian Medicine* in 2016. In addition, there is no indication in the published paper that a protocol was subject to ethical scrutiny by an appropriate committee within the authors’ institution.^18^ There are indeed questions over the ethical character of this study, especially with respect to ‘informed consent.’ Given that participants were aged between 14 and 18 years, many of them were minors and consent would be required both from parents and the children/young people themselves. There is no account provided, however, on whether consent was obtained. In fact, there are indications that girls may have been ‘required’ to participate given that the arts therapy sessions took place in school and during school hours (p. 53). More worryingly, for the girls with ‘externalising problems’ the authors state that:

… individuals in our study attended the sessions reluctantly and hence the required therapeutic relationship between the therapist and clients was rarely and barely established (p. 55).

This is an astonishing admission and surely undermines the credibility not only of the ‘externalising’ trial but the entire study.

A further omission in the paper is the lack of a CONSORT diagram, which would clearly specify the number of participants at every stage of the trial process – including the numbers of girls assessed in total before the selection of the sample, and the numbers lost to the trail during the intervention phase and afterwards.

#### Unclear Account of the Selection of Participants

A major concern with this study is the confusion that arises in the description given of how girls were identified as ‘internalising’ or ‘externalising.’ While it might appear straightforward from the summary given above, the precise procedures employed are not given, and inconsistencies arise in the text on the procedure, and between the account of the method and the data reported in the results section. To fully explain these points, it is necessary to quote at length from their account of the criteria used to identify the samples and the ASEBA:

Using targeted sampling, 30 students with internalising problems were selected and randomly assigned into experimental and control groups. Similarly, 30 students with externalising problems were chosen and randomly assigned to experimental and control groups. The selection process was based on students’ scores in the ASEBA considering test cut points (+2 standard deviations from the mean for internalising problems and + 1.3 standard deviations from the mean for externalising problems; p. 52).

This is straightforward and clear, but then they go on to say:

The main entry criteria the final sample for internalizing groups (experimental and control) was gaining a score equal or above +2 standard deviations in internalizing problems while having a score below +1.3 standard deviations in externalizing problems. The main entry criteria for externalizing groups (experimental and control) was gaining a score equal or above +1.3 standard deviations in externalizing problems while having a score below +2 standard deviations in internalizing problems (p. 52).

The picture then becomes even less clear in the following paragraph where it is claimed that scores from the ASEBA were ‘turned into T scores.’

To evaluate internalizing and externalizing problems, ASEBA was used. This self assessment questionnaire for adolescents includes 112 items and is normalized for 11 to 18 year-old individuals. The questions have been designed to evaluate emotional behavioral problems, social problems and desired social behaviors. The empirically based symptom scales include anxiety/depression, withdrawal/depression, somatoform complaints, rule-breaking and aggressive behaviours. The first three cases constitute internalizing problems and the last two constitute externalizing problems. The test scores range between 0–2. 0 = it does not apply to me; 1 = it is somehow and occasionally true for me; and 2 = it is completely and often true for me. Minimum and maximum scores for internalizing problems are 0 to 62, and for externalizing problems are 0 to 64. These scores were turned into T scores using a T-table. The clinical range for internalizing problems is T scores above 69 and for externalizing problems is T scores above 63 (p. 52).

To simplify the discussion, we will focus solely on the identification of girls with ‘internalising problems.’ The first thing to appreciate is that a cutoff point of +2 standard deviations means that the selected girls represent about the top 2.5% of the distribution if the ASEBA scores were approximately normally distributed. Thus, if they were selected by screening of the population of girls 14–18 in the school, a sample of 30 would imply that 1,200 girls were assessed but no details are given of the total number of girls screened as a basis for selection. However, the picture is more complicated because the internalising girls were not only at +2 standard deviations on the internalising scale but were also below +1.3 standard deviations on the externalising scale.

It appears, however, that the selection of the sample was based on normative data for the Achenbach scales as reference is made to the conversion of raw scores to T scores (standardised scores), with ‘the clinical range for internalising problems is T scores above 69.’ Unfortunately, this is entirely inconsistent with the statement that ‘minimum and maximum scores for internalising problems are 0–62’ – that is, the stated maximum score is below the stated cutoff point of 69.

The picture becomes more confused, when the reported data from the internalising trial are reported in Table 1 (p. 54), where it appears that the pre-test internalising mean for the total sample was 34.14. In other words, the average score for girls is in the middle of the stated range for the scale and is very much lower than the stated cutoff point for identifying ‘internalising problems.’

#### Problems With Power and Unclear Randomisation Procedures

To their credit, Bazargan and Pakdaman do consider the issue of power in their study. They refer to the use of ‘G-Power software’ to calculate a required sample size, given an estimated effect size of 0.25 and specified alpha and beta values. The figure that emerges is 30 participants (15 in each arm of the trial), which corresponds to the sample size previously specified in their paper. However, no justification is given of the anticipated effect size, which is small, and there is no discussion of estimates of the ‘minimum clinically important difference’, for the measures used. What is important surely, is an effect size which represents a meaningful change as assessed by the Achenbach scales. A further point is that they assume that the ‘correlation between repeated measures’ will be 0.75, but do not consider explicitly the possibility of regression to the mean. Given that the girls selected were above the 97th percentile on the score distribution, it is very likely that on retest, their scores would be lower, due to scale unreliability.

With respect to the process of randomisation of participants in the trials, Bazargan and Pakdaman say that ‘subject assignment into experimental and control group [was] accomplished by subjects names alphabetical sequence’ (p. 52), but this is unclear and questionable as means of undertaking randomisation.

#### Limited Description and Appropriateness of the Therapeutic Programme

Bazargan and Pakdaman devote two short paragraphs describing ‘the intervention package of Art therapy’ (p. 53). They say that the girls participated in six painting sessions during the school day in groups of between 3 and 15. This means that some sessions involved all the girls in the trials, whereas others involved smaller groups and so must have been repeated during the week. Bazargan and Pakdaman state that each session lasted one and a half hours, with an initial introduction, 45 min to an hour of painting and then ‘subjects had 15 min to talk with the therapist and other members about works, feelings, interests and events’ (p. 53). This short period of talking appears to have involved the whole group, and it is not clear what kind of benefits individual girls would have gained from such discussions. The implausibility of therapeutic benefits becomes more obvious when we consider the account given in the introduction of the putative processes involved in art therapy. They say, for example, that participants involved in art therapy can achieve ‘a feeling of security towards unpleasant memories of a traumatic event’ and can express ‘conflicts, fears and desires’ symbolically and thereby ‘regain a sense of personal agency’ (p. 52). Such processes may well happen in arts therapy undertaken individually, and over many sessions, with a sensitive therapist, but it is very hard to envisage how this would have happened in a short series of group sessions with adolescent girls in a school setting.

#### Problems in the Presentation and Analysis of Results

In the results section, Bazargan and Pakdaman explain that there was some ‘drop out of subjects through sessions and in the post-test phase’ for the internalising group, resulting in 14 girls in the experimental group and 13 in the control group. However, no data on attrition is reported for the externalising group. In addition, in Tables 1–3, which report the experimental results, no information is given on the final sample sizes. Figure 1 reporting on ‘the interactive effect of group and evaluation time on the internalising problems’ is also highly unsatisfactory, as no details are given of the scale on the *y*-axis (p. 53).

The statistical analysis performed on the pre-post data was ‘mixed analysis of variance’ and appears to be appropriately conducted, but some arcane details are given regarding tests applied to the data prior to this analysis, with reference to ‘the sphericity assumption and homogeneity of error variance,’ the lack of a need to perform the Mauchly test and the results from the Leven test^19^ which showed ‘that error variance values between groups in pretest and posttest were equal’ (p. 54).^20^ Such technicalities are confusing and of limited relevance, but a more serious problem is that close reading of this section shows that the statistics presented from the Levene test for both the internalising and externalising data are identical – with the same *F* and *p* values. This is entirely implausible and points to an error in reporting.

## Discussion

In this paper, we describe work undertaken as part of the process of conducting a systematic review of research on the role of arts engagement and art therapy with children and adolescents experiencing mental disorders (See ‘Footnote 1’). At the time of writing, we are working on identifying potentially suitable research papers to include in the review. We have searched electronic databases and screened abstracts and obtained full text versions of papers for further detailed scrutiny. We have also consulted previous relevant systematic reviews for studies in the broader filed on young people, arts engagement/art therapy and mental health.

At this stage, however, we have concerns regarding the quality of the research literature and previously published systematic reviews, and we have explored these concerns through an examination of one study of art therapy (Bazargan and Pakdaman, 2016) and its inclusion in three systematic reviews.

What emerges is the uncritical nature of the systematic reviews considered in which review teams were content to take the findings reported by Bazargan and Pakdaman at face value and repeat their conclusions, with little discussion and no serious scrutiny of their methods and results. While we consider only the Bazargan and Pakdaman paper in detail, examination of the tables in the reviews summarising studies included, raises a concern that the same may be true of every study included in each review.

### Further Comments on the Three Systematic Reviews

There is not enough space here to thoroughly compare and evaluate the three reviews considered in this paper. It would be a very time-consuming exercise. In the context of the present paper, it is also unnecessary as the central concern has been to consider the treatment of the Bazargan and Pakdaman paper in each of the reviews, which we have shown to be unsatisfactory. However, several critical points can be readily made about the reviews, and we do this in relation to the four ‘distinctive methodological features of systematic reviews’ as highlighted by Hammersley (2020): ‘exhaustive searching for relevant literature; explicit selection criteria regarding relevance and validity; and synthesis of relevant findings’ (p. 27).

All three reviews provide an account of their search strategies, inclusion/exclusion criteria and quality screening. These vary in detail and robustness and in the sources they utilise. What is striking about these reviews is the considerable diversity of the studies included, which leads the authors to divide the papers into categories dealing with different problems. In Ponomarenko et al. (2017), their report is structured as eight separate reviews according to the challenges facing the children/young people. Cohen-Yatziv and Regev (2019) follow the same strategy and report their review for five categories of issues addressed. Bosgraf et al. (2020), in contrast, organise their review according to the form of the art therapy and the role of the therapist.

The quality screening undertaken varies in each review, and the approach is either minimal (2017, 2019) or poorly reported (2020). In the 2017 and 2019 reviews the focus is simply on the design of the studies included and based on an uncritical assumption that RCTs provides ‘good’ evidence’.^21^ In the 2020 review, a more thorough quality assessment is described and is said to have been undertaken by two members of the review team independently and then agreed, but the details of the screening are not reported. Given that the authors of this review give the Bazargan and Pakdaman paper a ‘strong’ rating (they claim to have found no weaknesses), the care taken in conducting screening is called into question, and we can only wonder at the quality of studies they considered ‘weak.’ This raises the question of why the authors of the 2020 review did not exclude studies they judged to be ‘weak’ from their review.

In relation to ‘synthesis of relevant findings’ – the picture is also rather weak, as all reviews point to the heterogeneity of existing research studies, which makes any generalisations difficult. This is acknowledged explicitly by Cohen-Yatziv and Regev (2019) who indicate that a ‘meta-analysis’ would be ‘impossible’:

The findings described in this article emerge from the 13 studies that met the inclusion criteria. The decision to present these studies as a review rather than as a meta-analysis is due to the emergent nature of the field of art therapy. There is little research in the field, and the differences between studies and the indices are so great that it would have been impossible to produce a meta-analysis that would yield meaningful results (p. 103).

This statement is surely tantamount to saying that the corpus of studies is not amenable to synthesis through systematic review either.

A particularly important aspect of heterogeneity which is mentioned explicitly only by Ponomarenko et al. (2017), is the country where the study was conducted. The Bosgraf et al. (2020) paper is key to appreciating this challenge posed by studies coming from different countries, as it includes the largest number of studies. Of the 37 studies they include, a majority were undertaken in the United States (21), followed by Iran (7), Canada (2), Israel (2) and South Korea (2), and one each from South Africa, India and Germany. It is surely problematic to attempt to synthesise findings from studies conducted across such a wide range of different cultures.

### Further Factors Affecting the Quality of Reviews

Both Hammersley (2020) and Ioannidis (2016) offer interesting insights into why reviews can be unsatisfactory and uncritical.

Hammersley (2020) makes pertinent comments about the time involved in carrying out systematic reviews, and the need to balance resources devoted to each of these key elements. It may be that if disproportionate time is devoted to ensuring that the search is systematic and comprehensive, then less time is available for quality screening. Systematic reviews are undoubtedly onerous and require significant skills in critical reading, sustained concentration and careful negotiation within a review team to ensure that judgements made about the quality of studies (their strengths, limitations and weaknesses) and their findings are inter-subjectively agreed as accurate. As a great deal of time and effort is put into searching and selecting studies, and quality screening, reviewers may feel their work is done once the PRISMA flow chart is complete and that all that remains is to summarise the studies and attempt some form of narrative synthesis.

Ioannidis (2016) also makes interesting observations regarding the ‘vested interests’ of academics and the impact these have on the conduct of systematic reviews and meta-analyses. In his view:

Ideally, people who have no stake in the results should perform systematic reviews and meta-analyses, excluding not only those with financial conflicts of interest but even those who are content experts in the field. According to this line of argument, content experts can and should be consulted, but they should not be authors (p. 495).

His discussion raises a key question that needs to be asked of academics in the field of arts and health conducting reviews: Is the starting point of a review team one of ‘dispassionate enquiry and scepticism’ or is there a pre-established conviction that the arts have benefits for health and wellbeing? If the former, a review team may interrogate research methods and findings closely in the interests of establishing the truth or otherwise of claims made. If the latter, a review may be undertaken with the purpose of showcasing positive evidence. A further concern may be to advocate for supportive policy development, further funding for research and the practical implementation and wider scaling up of arts for health programmes.

This issue is a palpable potential source of bias in the Cohen-Yatziv and Regev (2019) review, as they acknowledge they are art therapists: ‘In the initial screening stage, both authors (who are certified art therapists) reviewed the abstracts to eliminate those that did not meet the research objectives’ (p. 102). The issue of ‘vested interests’ also extends to the process of acting as a reviewer for manuscripts submitted to a journal for publication. Regev, for example, is the sole reviewer named for the Bosgraf et al. (2020) review, which cites the Cohen-Yatziv and Regev (2019) review and an empirical paper by Regev and Guttmann (2005).

## Conclusion

Two conclusions emerge:

Firstly, the existing literature on young people, art therapy and health, included in the reviews considered, is so heterogeneous in multiple respects and limited in extent, that it is not amenable to systematic reviewing in a strict sense. And secondly, the reviews discussed are flawed due to forcing this literature through the ‘Procrustean bed’^22^ of systematic reviewing, compounded by a signal lack of robust critical scrutiny of the little evidence that does exit.

As a team, we need to consider whether we proceed with a systematic review of young people, arts engagement and mental health, as outlined in our current protocol; or whether we explore alternative models of reflecting on what can be learned from the existing body of evidence and practice. To assist us in addressing options, we will repeat the exercise described in this paper focusing on widely cited research on music therapy for children experiencing anxiety issues (Goldbeck and Ellerkamp, 2012), and the treatment of this research in no fewer than three recent meta-analyses (Geipel et al., 2018; Bear et al., 2020; Lu et al., 2021).

## Author Contributions

KG-H conceived and designed the systematic review of research on effects of arts-based programs for mental health in young people. SC, YI, LT-H, AB, and AKS-W contributed to the review design. AKS-W and KG-H search for studies through databases and systematic reviews and obtained full text papers and organised them alphabetically. SC search Google Scholar to identify citations of potentially relevant papers in subsequent publications and drafted and wrote the manuscript with support from other authors. KG-H and SC have screened abstracts for relevance. All authors were involved in the final drafting of the manuscript and provided critical feedback on the basis of their special areas of interest, which were incorporated into the final draft of the manuscript and approved the submitted version.

## Funding

KG-H and AKS-W were supported by Land Salzburg. The open access publication costs for article will be covered by University Mozarteum Salzburg. The funders had no role in the conceptualisation, design, literature searches, analysis, interpretations, preparation of the manuscript or decision to publish.

## Conflict of Interest

The authors declare that the research was conducted in the absence of any commercial or financial relationships that could be construed as a potential conflict of interest.

## Publisher’s Note

All claims expressed in this article are solely those of the authors and do not necessarily represent those of their affiliated organizations, or those of the publisher, the editors and the reviewers. Any product that may be evaluated in this article, or claim that may be made by its manufacturer, is not guaranteed or endorsed by the publisher.

## Footnotes

^1^ https://www.crd.york.ac.uk/prospero/display_record.php?RecordID=193283

^2^Critical discussions of systematic reviewing can be found beyond medicine and the health sciences in the field of International Development (Mallett et al., 2012) and education (MacLure, 2005; Hammersley, 2020, see below).

^3^We are not claiming here that all systematic reviews and meta-analyses are flawed. Properly conducted, focused reviews and meta-analyses can serve an important function in summarising developments in a field of research, guiding improvements in healthcare and providing guidance for needed future research.

^4^A 37-item assessment instrument to assess the methodological quality of systematic reviews, building upon previous tools, empirical evidence and expert consensus. See Shea et al. (2007).

^5^We have been unable to find any information online on the character and curriculum of Iranian ‘art schools.’ We assume, however, that such schools promote Islamic values, are single-sex and train students in traditional Iranian arts and crafts (see: https://surfiran.com/arts-crafts-iran/).

^6^Note that these three symptom clusters are distinct, but it can be assumed given the cut off point for inclusion of ‘internalising’ girls in the study, that they reported all three forms of difficulty.

^7^Note that these two symptom clusters appear very different, and girls may be ‘rule-breaking’ without being ‘aggressive’ and vice versa. However, given the cut off point for inclusion of ‘externalising’ girls, it can be assumed that they reported both forms of behaviour.

^8^Details of the Scientific Maryland Scale are given in Appendix A of the report. The original source for the scale, oddly, is an American report on research in preventing crime: Sherman et al. (1998). Preventing crime: What works, what does not, what’s promising. Report to the U.S. Congress, Washington, DC.

^9^No details are given of the process and reliability of rating sources against this scale.

^10^An explanation of this rating may be that Pnomarkenko et al. regard the Bazargan and Pakdaman study as ‘quasi-experimental’ (p. 77). Bazargan and Pakdaman confusingly describe their study as ‘semi-experimental’ most likely in referring to the comparison made between the effects of art therapy for the internalising and externalising groups of girls involved in the study. Within these separate samples, however, girls were randomly assigned and so, in fact, the paper reports two separate trials taking place in parallel.

^11^Note the language used. Why ‘child-aged clients’ rather than just ‘children?’

^12^The research studies identified are almost identical to those located by Ponomarenko et al. (2017).

^13^Many more studies are identified compared with the two previous reviews as more databases are searched and a longer time span is considered.

^14^The quality assessment tool can be found here: EPHPP – McMaster Evidence Review & Synthesis Centre (merst.ca).

^15^This is a considerable weakness in Bosgraf et al.’s review. Despite employing a more appropriate quality assessment tool than both earlier reviews, it is disappointing not to see the details of their assessment. Bazargan and Pakdaman, for example, do not discuss ‘blinding’ and as we will see below there are substantial problems with ‘intervention integrity’ and ‘analysis.’

^16^The term ‘intervention’ is considered inappropriate in the context of creative arts therapies as it implies an action ‘done to’ participants, rather than a process of active engagement by participants. The broader principle is ‘Nihil de nobis, sine nobis.’ See: https://en.wikipedia.org/wiki/Nothing_About_Us_Without_Us

^17^For a discussion of the requirements of trial registration see: https://www.biomedcentral.com/getpublished/writing-resources/trial-registration

^18^Current guidance for publication in the *Archives of Iranian Medicine* specifies that controlled trials should be registered and subject to review: http://www.aimjournal.ir/InstructionsforAuthors

^19^The authors refer to the ‘Leven test’ when they mean the test developed by Levene to test for equality of variance in two groups: https://en.wikipedia.org/wiki/Levene%27s_test

^20^This is poorly expressed by the authors, since the test can only serve to reject the null hypothesis, rather than support it.

^21^It should be acknowledged, however, that Ponomarenko et al. (2017) do provide a comprehensive listing of the weaknesses of the studies they review in their discussion (see p. 121 onwards).

^22^ https://www.merriam-webster.com/dictionary/procrustean%20bed
